# Olive Fruit Extracts Supplement Improve Antioxidant Capacity *via* Altering Colonic Microbiota Composition in Mice

**DOI:** 10.3389/fnut.2021.645099

**Published:** 2021-04-06

**Authors:** Mengyu Wang, Shunfen Zhang, Ruqing Zhong, Fan Wan, Liang Chen, Lei Liu, Bao Yi, Hongfu Zhang

**Affiliations:** ^1^State Key Laboratory of Animal Nutrition, Institute of Animal Science, Chinese Academy of Agricultural Sciences, Beijing, China; ^2^College of Pastoral Agriculture Science and Technology, Lanzhou University, Lanzhou, China

**Keywords:** olive extracts, antioxidant capacity, gut microbiota, oxidative stress, anti-inflammatory capacity, hydroxytyrosol

## Abstract

Oxidative stress, one of the most common biological dysfunctions, is usually associated with pathological conditions and multiple diseases in humans and animals. Chinese olive fruit (*Canarium album* L.) extracts (OE) are natural plant extracts rich in polyphenols (such as hydroxytyrosol, HT) and with antioxidant, anti-hyperlipidemia, and anti-inflammatory potentials. This study was conducted to investigate the antioxidant capacity of OE supplementation and its related molecular mechanism in mice. Mice (25.46 ± 1.65 g) were treated with 100 mg/kg body weight (BW) OE or saline solution for 4 weeks, and then the antioxidant and anti-inflammatory capacities of mice were examined. The results showed that OE supplement significantly increased the serum antioxidative enzyme activities of total antioxidant activity (T-AOC), superoxide dismutase (SOD), glutathione peroxidase (GSH-Px), and catalase and decreased the serum malondialdehyde (MDA) level, indicating that OE treatment enhanced the antioxidant capacity in mice. qPCR results showed that the transcriptional expression of antioxidant *SOD1, CAT, Gpx1*, and *Gpx2* were significantly down-regulated in the small intestine (jejunum and ileum) after OE administration. Meanwhile, OE treatment significantly decreased the T-AOC and increased the MDA level in the small intestine. Furthermore, OE administration dramatically reduced the mRNA expression of pro-inflammatory cytokines (TNF-α and IL-1β), which confirmed its antioxidant and anti-inflammatory capacities with OE administration. Using amplicon sequencing technology, 16S rRNA sequencing results showed that OE supplement significantly increased the colonic *Firmicutes*/*Bacteroidetes* ratio, which also had a negative correlation with the serum MDA level and positively correlated with serum GSH-Px activity through Pearson correlation analysis. Besides that, *Alloprevotella* was negatively correlated with serum T-AOC. *Colidextribacter* was positively correlated with serum MDA and negatively correlated with serum T-AOC, SOD, and GSH-Px levels. In summary, this study showed that treatment with 100 mg/kg BW polyphenol-rich OE could alter colonic microbiota community, which was strongly associated with improved antioxidant capacity in mice.

## Introduction

Oxidative stress is regarded as a result of the imbalance of oxidants and antioxidants, which can cause damage to important cellular macromolecules, such as DNA, lipid, and protein, and, in turn, lead to toxicity, chronic inflammation, and diseases, acting as a serious threat to animal and human health (1–3). In animal husbandry, oxidative stress is commonly considered to be associated with various pathological conditions and can severely damage productivity and livestock product quality and even lead to death (4, 5). Similarly, oxidative stress is also an important factor for the progression of human diseases and body disorders, including metabolic diseases and inflammation-related diseases, such as inflammatory bowel disease and diabetes (6–8). Moreover, the overproduction of reactive oxygen species and reactive nitrogen species during oxidative stress can cause inflammatory responses by activating the related signal transduction pathways (9, 10).

Polyphenols are natural compounds present in plants with numerous biological activities, which have been proposed to be useful as adjuvant therapy for their potential antioxidant effect, associated with the anti-inflammatory activity (11). Olive extracts, as one of the important natural plant extracts, have been extensively explored for their potential antioxidant properties (12, 13). The main bioactive component of olive extracts are polyphenols, which are thought to be responsible for their wide range of biological activities. Increasing evidence has indicated that olive extracts rich in polyphenolic compounds have powerful antioxidant and anti-inflammatory effects in mammalian cells, rats, and humans (14–17). Administration of olive oils high in phenolic compounds decreased malondialdehyde (MDA) levels in urine and increased plasma glutathione peroxidase (GSH-Px) activity in a dose-dependent manner in men (18). Olive leaf extract could enhance antioxidation capacity in the liver of aged mice by inducing a decrease in the MDA level and an increase in glutathione (GSH) level (19). Olive pomace extracts supplement followed with increased total antioxidant activity (T-AOC) was shown in the study of A. De Bruno et al. (20). In addition, some studies have shown that olive extracts have anti-bacterial and anti-inflammation effects (21–23). It is well-established that these beneficial health properties of olive extracts are related to one of the polyphenolic compounds named hydroxytyrosol (HT) (24–26). Studies in mammalian cells have demonstrated that HT can exert potential effects against oxidative stress and inflammation (24, 27). Further mechanism research showed that HT alleviated oxidative stress by decreasing the production of oxygen species (24). Thus, olive extracts enriched with various polyphenols (especially HT) may be an effective prevention against disorders related to oxidative stress.

Concerning the metabolism of olive extracts, particularly the olive bioactive component polyphenols, growing evidence has demonstrated that only small amounts of ingested polyphenols can be absorbed in the small intestine and enter the systemic circulation (28, 29). Most remaining polyphenols reach into the large intestine, where they can be metabolized by gut microbiota (28, 29). The colonic microbiota, therefore, plays a key role in the metabolism of polyphenols. A study showed that olive administration could alleviate hypercholesterolemia by reducing the relative abundance of *Lactobacilli* and *Ruminococcus* in the human gut microbiota (30). Extra virgin olive oil supplementation increased the gut microbiota diversity and decreased the relative abundance of *Firmicutes* in mice (31). In addition, another study showed that olive leaf extract can counteract the ecological disorders associated with obesity by altering the colonic microbial community in mice (32). These studies have suggested that olive oil and olive leaf phenolic compounds can induce changes in gut microbial composition and alter its metabolism in mice and humans with metabolic diseases.

However, whether oral administration of Chinese olive fruit (*Canarium album* L.) extracts (OE) could improve colonic microbiota and whether colonic microbiota is a remarkable mechanism further involved in antioxidant and anti-inflammatory effects of OE remain to be elucidated. We here, therefore, investigated the effects of OE on the levels of antioxidant indicators, the expressions of antioxidant enzymes and inflammatory cytokines in the intestine, and the colonic microbiota composition to explore its underlying molecular mechanism.

## Materials and Methods

### Reagents, Mice, and Ethics

The OE was purchased from Shanghai Huahan Biotechnology Co., Ltd. (Shanghai, China), and it was composed of 10 wt% HT as checked by high-performance liquid chromatography. Specifically, an Acquity UPLC BEH C18 (1.7 μm, 2.1 × 50 mm) column was used. The binary mobile phase consists of two different formic acid solutions running in a linear gradient, and detection is carried out with UV–vis at 278 nm. Quantification was performed by the external standard method with tyrosol and HT reference standards. Then, the concentrations of these compounds were calculated using the response factor of HT reference standard. Three-week-old female ICR mice were purchased from Peking University Health Science Center (Beijing, China). The mice were maintained in a 12-h light/dark cycle, with free access to diet and water. All procedures used in this experiment were approved by the Experimental Animal Welfare and Ethical Committee of the Institute of Animal Science, Chinese Academy of Agricultural Sciences (no. IAS2020-86).

### Mice Experiment and Sampling

After 1-week acclimatization, the mice (25.46 ± 1.65 g) were divided into two groups (*n* = 12 per group). The OE supplement (OE) group was treated with 100 mg/kg body weight OE (prepared fresh in distilled water before gavage), and the control (Con) mice received the same volume of distilled water every day *via* oral gavage. Body weight and feed intake of the mice were measured weekly. At the end of day 28, blood samples were collected by orbital blooding, and then the mice were killed by cervical dislocation. The jejunum and ileum tissues were quickly removed and frozen in liquid nitrogen for further analysis. For histopathology examinations, part of the jejunum and ileum were cut and fixed in 4% paraformaldehyde. Colonic digesta were collected for 16S rRNA sequencing and short-chain fatty acid analysis.

### Serum Oxidant and Antioxidant Marker Analyses

Serum was obtained by centrifugation at 1,000 *g* for 15 min under 4°C and stored in aliquots at −80°C. The activities of total antioxidant capacity (T-AOC), glutathione peroxidase (GSH-Px), catalase (CAT), and superoxide dismutase (SOD) and the level of malondialdehyde (MDA) and inflammatory cytokines (TNF-α, IL-1β, IL-6, and IFN-γ) were measured with corresponding assay kits (Nanjing Jiancheng Bioengineering Institute, Nanjing, China) following the manufacturers' instructions.

### Intestinal Morphology Examination

Proximal jejunum and distal ileum sections were used for histologic examination. They were fixed with 4% paraformaldehyde–phosphate-buffered saline overnight, then dehydrated, and embedded in paraffin blocks. After that, a section of 5 μm was cut and mounted on slides. The sections were further deparaffinized and hydrated and then stained with hematoxylin-eosin (H&E) for microscopy. Microphotographs were taken with a DM300 microscope (Leica, Germany). Villus length and crypt depth were performed using Image J software. A minimum of 20 well-orientated villi and associated crypts from at least seven different fields per animal were measured.

### RNA Extraction and Quantitative Real-Time Polymerase Chain Reaction Analysis

Total RNAs from jejunum and ileum samples were isolated using Trizol (Invitrogen, USA) reagent and then treated with DNase I (Invitrogen, USA) according to the instruction of the manufacturer. The concentration of each RNA sample was quantified using NanoDrop 2000 (Nanodrop Technologies, USA). Before reverse transcription, possible contaminations from genomic DNA were eliminated using a PrimeScript RT reagent kit (Takara, Japan). cDNA was synthesized using PrimeScript Enzyme Mix 1, RT Primer Mix, and 5 × PrimerScript Buffer 2 (Takara, Dalian, China). Reverse transcription was conducted at 37°C for 15 min and 85°C for 5 s. Gene-specific prime sequences (Table 1) were designed using Primer 5.0 software and synthesized by Sangon Biotech Co., Ltd (Shanghai, China). Real-time PCR was performed according to the manufacturer's instructions. Briefly, 1 μl cDNA template was added to a total volume of 10 μl containing 5 μl KAPA SYBR FAST qPCR Master Mix Universal, 0.4 μl PCR forward primer, 0.4 μl PCR reverse primer, 0.2 μl ROX low, and 3 μl PCR-grade water (Kapa Biosystems, Beijing, China). We used the following protocol: (i) enzyme activation (3 min at 95°C), (ii) an amplification and quantification program consisting 40 of repeated cycles (3 s at 95°C and 34 s at 60°C), and (iii) a melting curve program (15 s at 95°C, 1 min at 60°C, and 15 s at 95°C). Relative expression was calculated between the control group and treatment group by 2^−ΔΔCt^ method, where Δ*C*_*t*_ = *C*_*t*_ (Target)–*C*_*t*_ (β-actin). β-actin was chosen as a housekeeping gene to normalize target gene transcript level.

**Table 1:** Primers used for qPCR assay.

**Gene**	**Accession no**.	**Sequence (5^**′**^-3^**′**^)**
β-actin	NM_007393.5	F: TGTCCACCTTCCAGCAGATGT
		R: GCTCAGTAACAGTCCGCCTAGAA
SOD1	NM_011434.2	F: GTGAACCAGTTGTGTTGTC
		R: ATCACACGATCTTCAATGGA
CAT	NM_009804.2	F: TCAGGTGCGGACATTCTA
		R: ATTGCGTTCTTAGGCTTCT
GPx1	NM_001329527.1	F: ATCAGTTCGGACACCAGA
		R: TTCACTTCGCACTTCTCAA
GPx2	NM_030677.2	F: GTGGCGTCACTCTGAGGAACA
		R: CAGTTCTCCTGATGTCCGAACTG
TNF-α	NM_013693.3	F: CATCTTCTCAAAATTCGAGTGACAA
		R: TGGGAGTAGACAAGGTACAACCC
IL-1β	NM_008361.4	F: TTCAGGCAGGCAGTATCA
		R: CCAGCAGGTTATCATCATCA

*F, forward; R, reverse*.

### Bacterial 16S rRNA Gene Sequencing and Analysis

Total genome DNA from colonic digesta was extracted using QIAamp DNA Stool Mini Kit (Qiagen, Germany); then, DNA concentration and purity was monitored on 1% agarose gels. The V3–V4 region of the bacterial 16S ribosomal RNA gene was amplified using a specific primer (338F, 5′-ACTCCTACGGGAGGCAGCAG-3′; 806R, 5′-GGACTACHVGGGTWTCTAAT-3′). Amplicons were detected using 2% agarose gel electrophoresis and purified using the AxyPrep DNA gel extraction kit (Axygen Bioscience, CA, USA) according to the manufacturer's instructions. After having been quantified and purified, the amplicons were sequenced using Illumina MiSeq platform (Illumina, San Diego, CA, USA) at Majorbio Bio-Pharm Technology Co., Ltd. (Shanghai, China) according to standard protocols. The raw reads were deposited into the NCBI Sequence Read Archive database (Accession Number: PRJNA681369). The sequences were analyzed and assigned to operational taxonomic units (OTUs; 97% identity). Alpha diversity was analyzed using QIIME (Version 1.7.0), which included the calculation of ACE, Chao 1, Shannon, and Simpson indices. Beta diversity was estimated by computing the unweighted Unifrac distance and visualized using principal coordinates analysis (PCoA).

### Short-Chain Fatty Acid Analysis

For short-chain fatty acid (SCFA) analysis, frozen colonic digesta samples (100 mg) were weighed into 1.5-ml centrifuge tubes and mixed with 1 ml ddH_2_O, homogenized, and centrifuged at 10,000 rpm for 10 min under 4°C. A mixture of the supernatant fluid and 25% metaphosphoric acid solution (0.9 and 0.1 ml, respectively) was vortexed for 1 min and centrifuged at 1,000 rpm for 10 min under 4°C after letting it stand in a 1.5-ml centrifuge tube at 4°C for over 2 h. The supernatant portion was then filtered through a 0.45-μm polysulfone filter and analyzed using Agilent 6890 gas chromatography (Agilent Tecnologies, Inc., Palo Alto, CA, USA).

### Statistical Analysis

All statistical analyses were performed by using Student's *t*-test (SPSS 21 software). Pearson correlation analysis between the *Firmicutes*/*Bacteroidetes* ratio and serum antioxidant indicators (T-AOC, SOD, GSH-Px, CAT, and MDA) was carried out using GraphPad Prism 7.0. Data are expressed as mean ± SEM. *P*-value < 0.05 was considered significant.

## Results

### Effects of OE Supplement on Body Weight and Feed Intake

**Figure 1:**
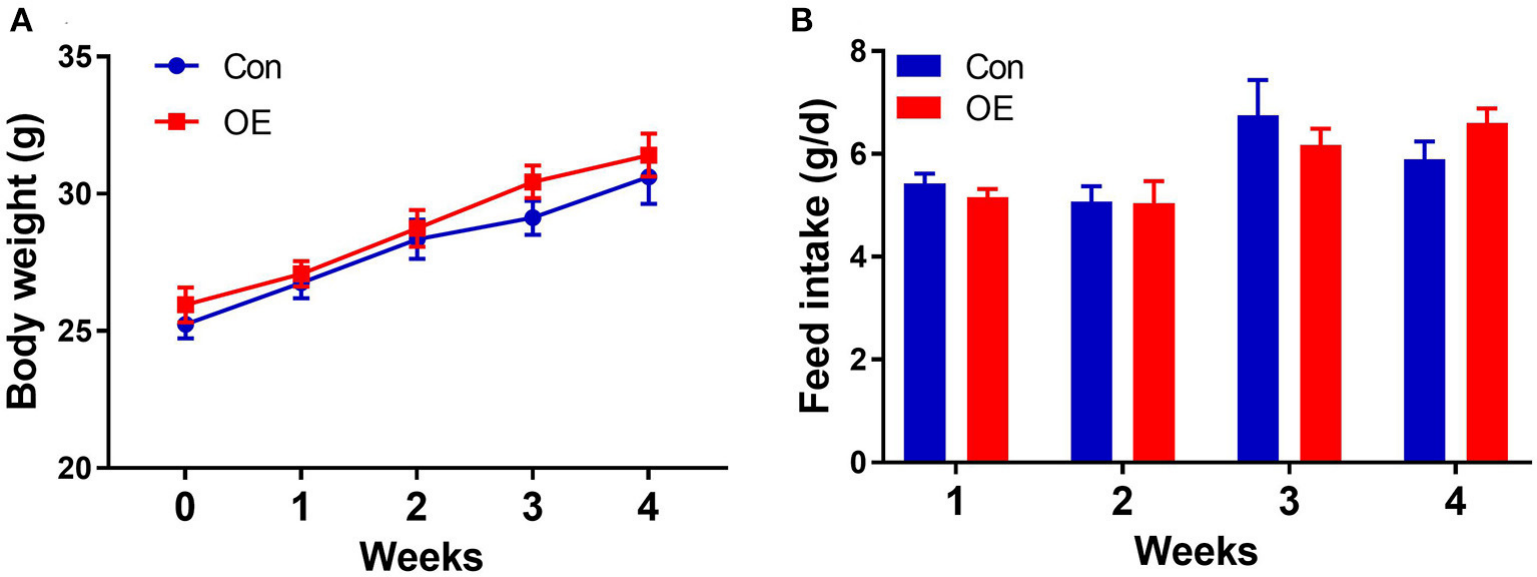
Effects of olive fruit (*Canarium album* L.) extracts on body weight and feed intake. **(A)** Body weight. **(B)** Average daily feed intake. Data were expressed as mean ± SEM.

Oral administration with OE for 4 weeks had no significant effects on the body weight (*P* > 0.05) and average daily feed intake in mice compared with the mice in the Con group (*P* > 0.05; Figure 1).

### OE Supplement Enhanced the Serum Antioxidant Capacity in Mice

**Figure 2:**
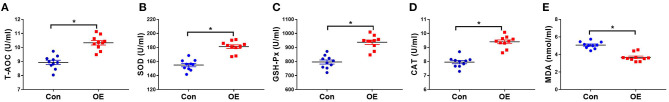
Effects of olive fruit (*Canarium album* L.) extracts on the serum antioxidant indicators. **(A)** T-AOC, **(B)** SOD, **(C)** GSH-Px, **(D)** CAT, **(E)** MDA. Data were expressed as mean ± SEM. *P < 0.05.

The activities of oxidant–antioxidant enzyme and MDA levels are sensitive indicators for oxidative stress. To determine whether OE affects antioxidant capacity, serum oxidant–antioxidant enzyme activities of T-AOC, SOD, GSH-Px, CAT, and MDA levels were analyzed using test kits. Figure 2 shows that OE treatment significantly increased the T-AOC (*P* < 0.05), increased the activities of SOD (*P* < 0.05), GSH-Px (*P* < 0.05), and CAT (*P* < 0.05), and decreased the level of MDA (*P* < 0.05).

### Effects of OE Supplement on Intestinal Morphology

**Figure 3:**
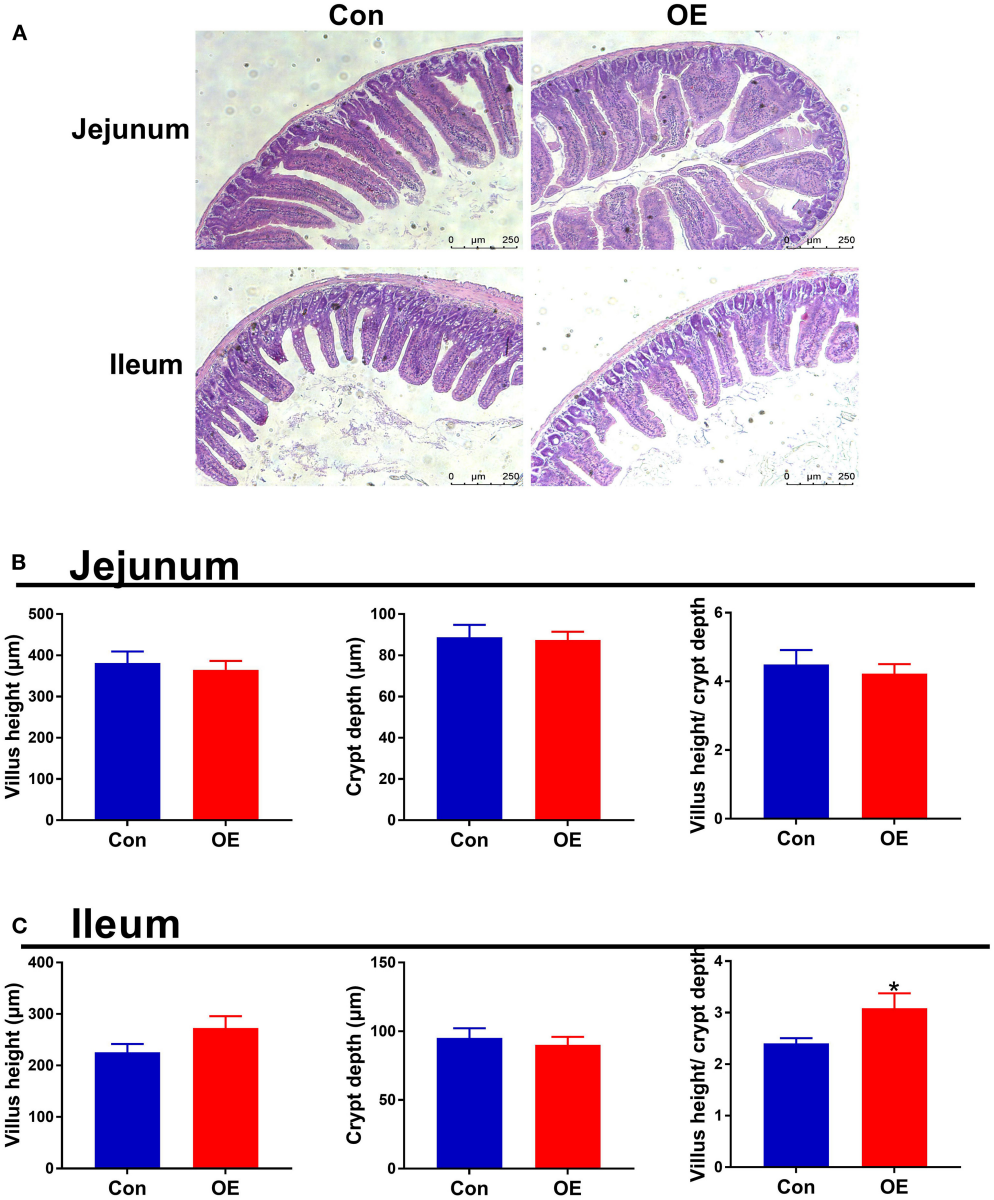
Effects of olive fruit (*Canarium album* L.) extracts on jejunal and ileal morphology. **(A)** Representative images of H&E staining in the jejunum and ileum. **(B)** Jejunal villus height, crypt depth, villus height/crypt depth. **(C)** Ileal villus height, crypt depth, villus height/crypt depth. Data were expressed as mean ± SEM. *P < 0.05.

Intestinal morphology was examined with H&E staining. The villus height, crypt depth, and villus height/crypt depth ratio were measured (Figure 3). In the jejunum, treatment with OE had no significant effects on the villus height, crypt depth, and villus height/crypt depth ratio in mice (*P* > 0.05; Figure 3B). In the ileum, the villus height and crypt depth had no significant difference between the two groups (*P* > 0.05; Figure 3C). However, the villus height/crypt depth ratio was significantly higher in the OE group than in the Con group (*P* < 0.05; Figure 3C).

### OE Altered Small Intestinal Antioxidant Capacity

**Figure 4:**
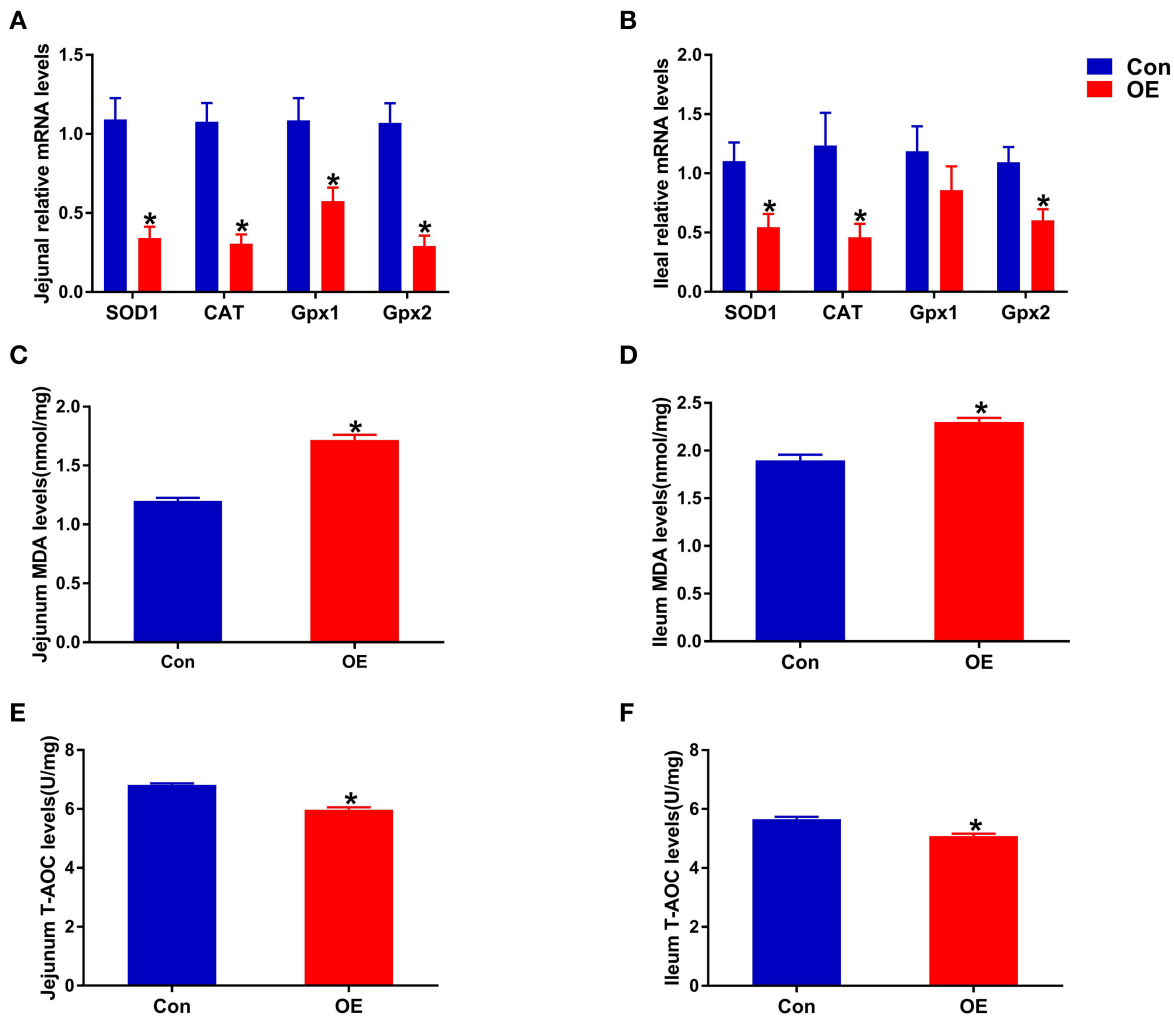
Effects of olive fruit (*Canarium album* L.) extracts on the jejunal and ileal antioxidant capacities. **(A)** Jejunum mRNA expression levels of antioxidant enzymes. **(B)** Ileum mRNA expression levels of antioxidant enzymes. **(C)** Jejunum MDA levels. **(D)** Ileum MDA levels. **(E)** Jejunum T-AOC levels. **(F)** Ileum T-AOC levels. Data were expressed as mean ± SEM. *P < 0.05.

Next, we analyzed the mRNA expression of genes associated with antioxidant capacity in the ileum and jejunum, including *SOD1, Gpx1, Gpx2*, and *CAT* to examine the molecular mechanism of OE administration in enhancing antioxidant capacity. In the jejunum, compared with the Con group, OE treatment significantly down-regulated the mRNA expression of *SOD1, CAT, Gpx1*, and *Gpx2* (*P* < 0.05; Figure 4A). Similarly, in the ileum, the mRNA expression of *SOD1, CAT*, and *Gpx2* was markedly lower in the OE group than in the Con group (*P* < 0.05; Figure 4B). Besides that, the results showed that, in the ileum and jejunum, the MDA level was significantly higher (*P* < 0.05), while the T-AOC was significantly lower in the OE group compared with that in the Con group (*P* < 0.05; Figures 4C–F).

### OE Supplement Altered the Expression and Levels of Pro-inflammatory Cytokines in the Small Intestine

**Figure 5:**
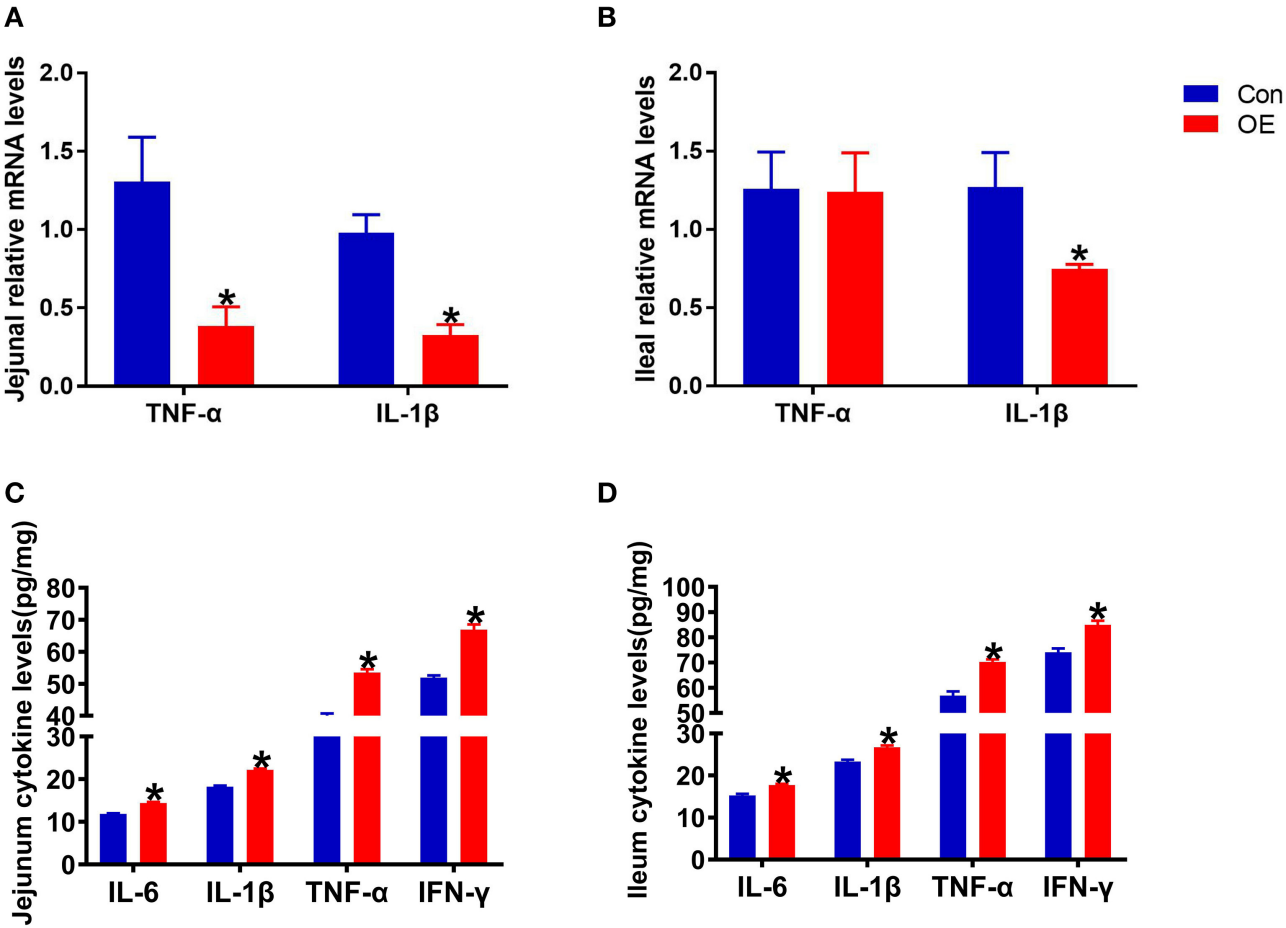
Effects of olive fruit (*Canarium album* L.) extracts on the jejunal and ileal pro-inflammatory cytokines. **(A)** Jejunal relative mRNA levels of cytokines. **(B)** Ileal relative mRNA levels of cytokines. **(C)** Jejunal cytokine levels. **(D)** Ileal cytokine levels. Data were expressed as mean ± SEM. *P < 0.05.

Oxidative stress is often involved in inducing inflammatory responses. Thus, the anti-inflammatory capacity of OE was also tested by analyzing the mRNA expression of pro-inflammatory cytokines (TNF-α and IL-1β) in the intestine. The results showed that, in the jejunum, oral administration of OE markedly down-regulated the mRNA levels of TNF-α and IL-1β (*P* < 0.05; Figure 5A). OE administration showed a likely significant decrease in IL-1β mRNA expression in the ileum compared with the Con group (*P* < 0.05; Figure 5B). However, the ELISA results showed that, in the jejunum and ileum, the pro-inflammatory cytokine (IL-6, IL-1β, TNF-α, and IFN-γ) levels were significantly higher in the OE group than in the Con group (*P* < 0.05; Figures 5C,D).

### OE Supplement Altered the Composition of Colonic Microbiota Community

**Figure 6:**
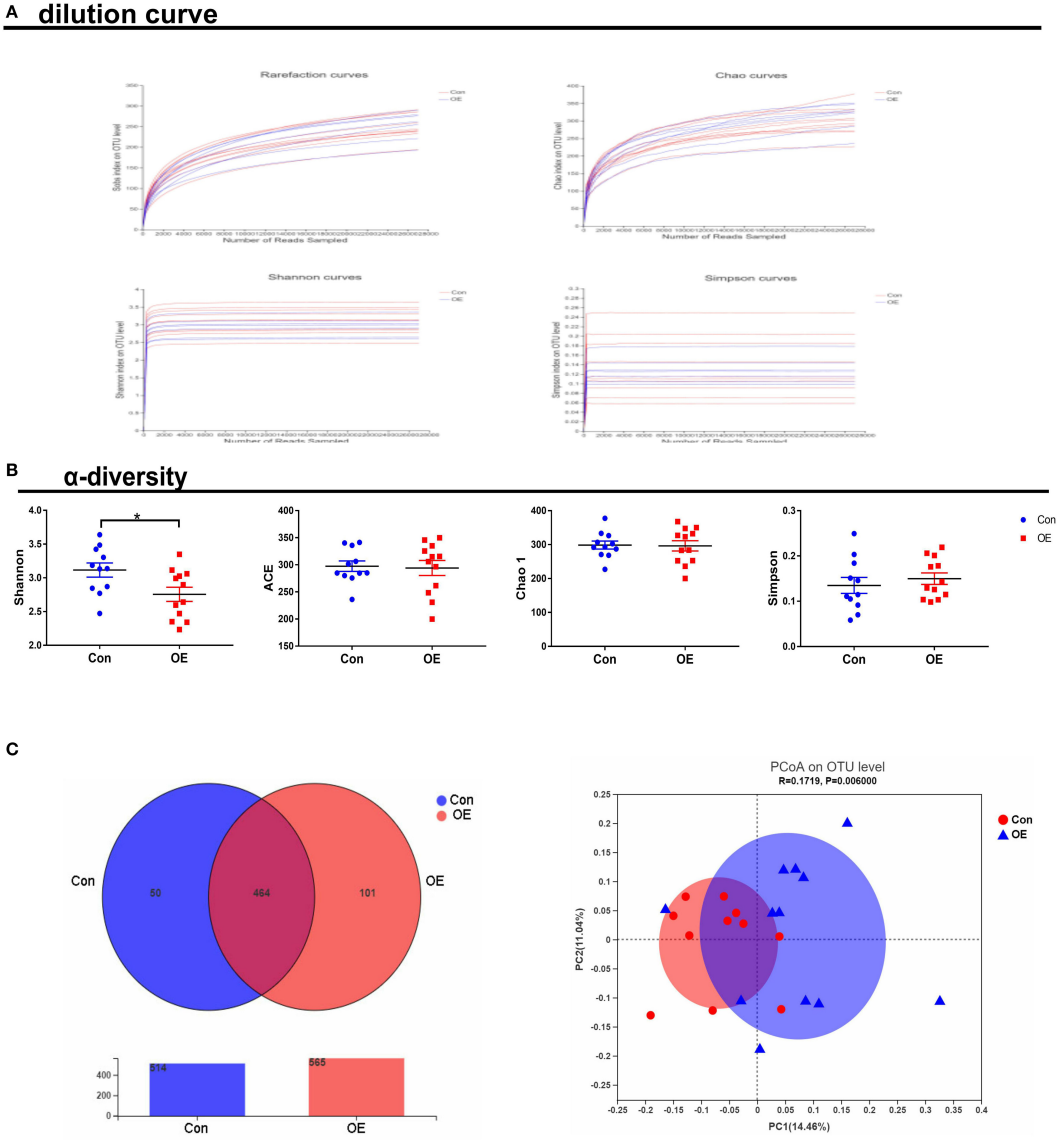
Effects of olive fruit (*Canarium album* L.) extracts on the colonic microbial diversity. **(A)** Dilution curve. **(B)** α-diversity. **(C)** Venn diagram. Data were expressed as mean ± SEM.

To study the effect of OE supplementation on large intestinal microbiota composition, the colonic chyme microflora was analyzed by sequencing V3 + V4 regions of 16S rRNA genes. After removing the low-quality sequences, a total of 1,184,959 clean tags were clustered into OTUs based on 97% identity. The dilution curves showed that the end of the curve tends to be flat, indicating that the amount and depth of high-throughput sequencing data is reliable (Figure 6A). To identify the microbial α-diversity, ACE and Chao 1 indexes were examined for the community richness, and Shannon and Simpson were examined for the community diversity. As shown in Figure 6B, OE treatment significantly decreased the Shannon index (*P* < 0.05), while it had a little effect on the ACE, Chao 1, and Simpson indexes compared to the Con group. The Venn diagram shows that there are 464 common OTUs between the Con and OE groups. Meantime, the Con and OE groups contained individual 50 and 101 OTUs, respectively (Figure 6C). To further understand the microbial composition between the Con and OE groups, we evaluated β-diversity using PCoA based on unweighted Unifrac. The results showed that the microbial community structure in the OE group significantly differed from that in the control group (Figure 6C).

**Figure 7:**
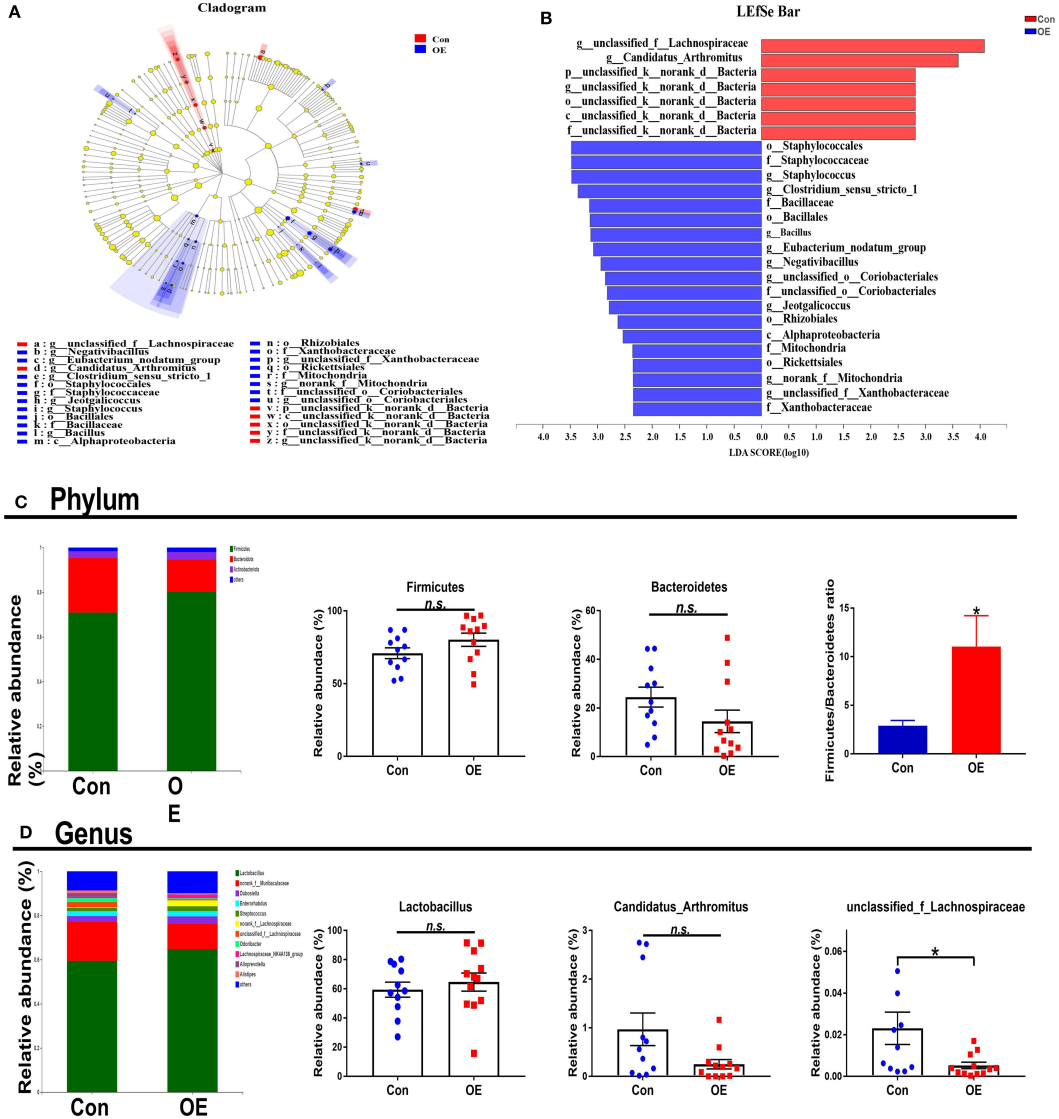
Effects of olive fruit (*Canarium album* L.) extracts on the colonic microbial composition. **(A,B)** LEfSe analysis. **(C)** Phylum level. **(D)** Genus level. Data were expressed as mean ± SEM. *P < 0.05; n.s., not statistically significant.

The overall microbial composition in the Con and OE groups differed at the phylum and genus levels. Linear discriminant analysis effect size (LEfSe) analysis was performed to evaluate the differentially expressed bacteria. Of note is the fact that *Staphylococcales* and *Bacillaceae* were shown to be enriched in the OE treatment group (Figures 7A,B). The relative abundance results showed that, at the phylum level, OE supplement notably enhanced the *Firmicutes/Bacteroidetes* ratio (*P* < 0.05), while it did not affect the relative abundance of *Firmicutes* and *Bacteroidetes*, respectively (*P* > 0.05; Figure 7C). At the genus level, OE tended to decrease the relative abundance of *Candidatus_Arthromitus*, but the difference was not significant (*P* > 0.05). However, the relative abundance of *unclassified_f_Lachnospiraceae* was significantly lower in the OE group than that in the control group (*P* < 0.05; Figure 7D).

### The Association Analysis Between OE Supplement-Induced Alterations in Colonic Microbiota and Serum Antioxidant Capacity

**Figure 8:**
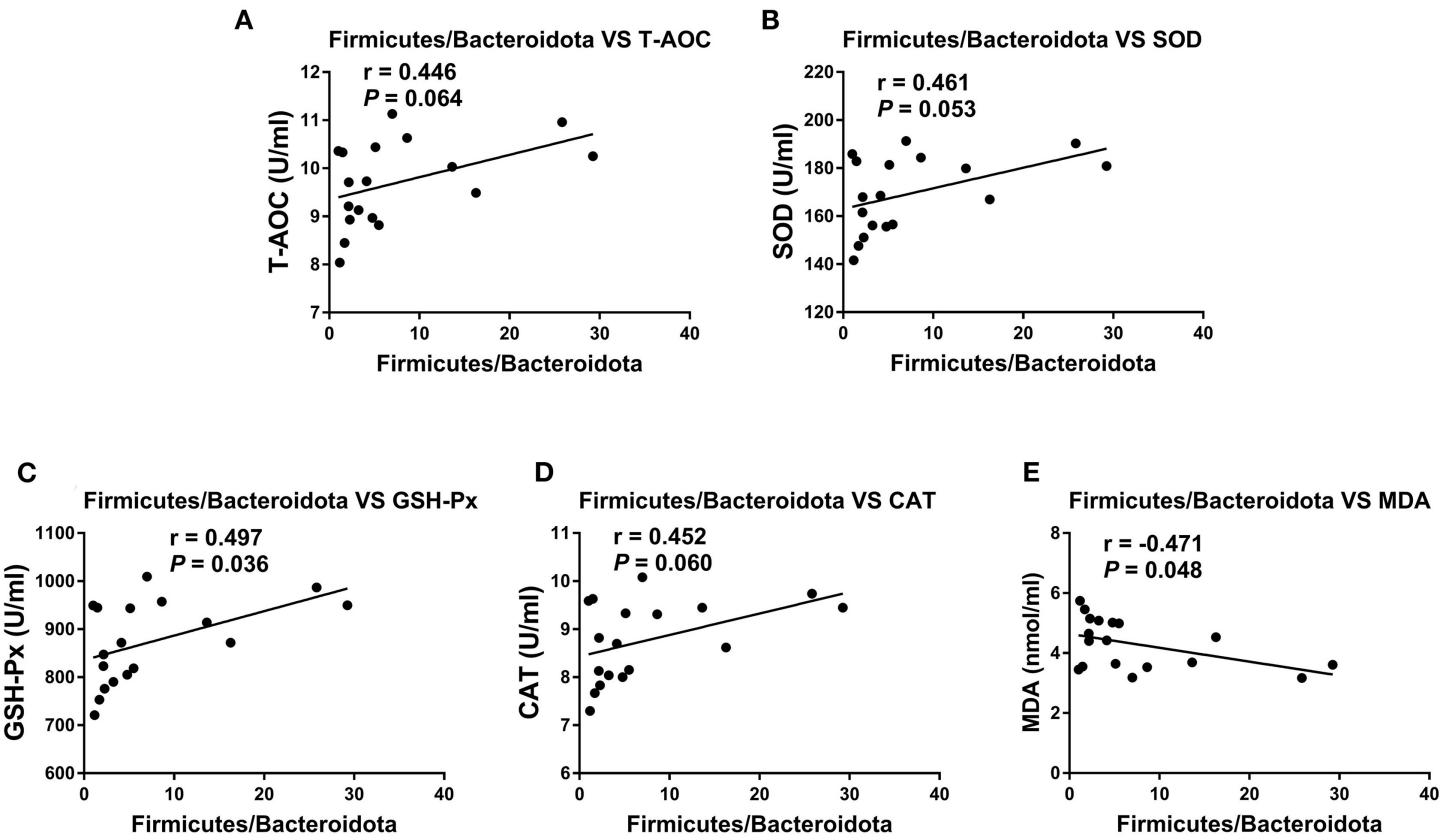
Pearson correlation analyses between the colonic *Firmicutes*/*Bacteroidetes* ratio and serum antioxidant indicators and liver and kidney mRNA expression levels of TNF-α and IL-1β. **(A)** Correlation analyses between the *Firmicutes*/*Bacteroidetes* ratio and serum T-AOC. **(B)** Correlation analyses between the *Firmicutes*/*Bacteroidetes* ratio and serum SOD. **(C)** Correlation analyses between the *Firmicutes*/*Bacteroidetes* ratio and serum GSH-Px. **(D)** Correlation analyses between the *Firmicutes*/*Bacteroidetes* ratio and serum CAT. **(E)** Correlation analyses between the *Firmicutes*/*Bacteroidetes* ratio and serum MDA.

**Figure 9:**
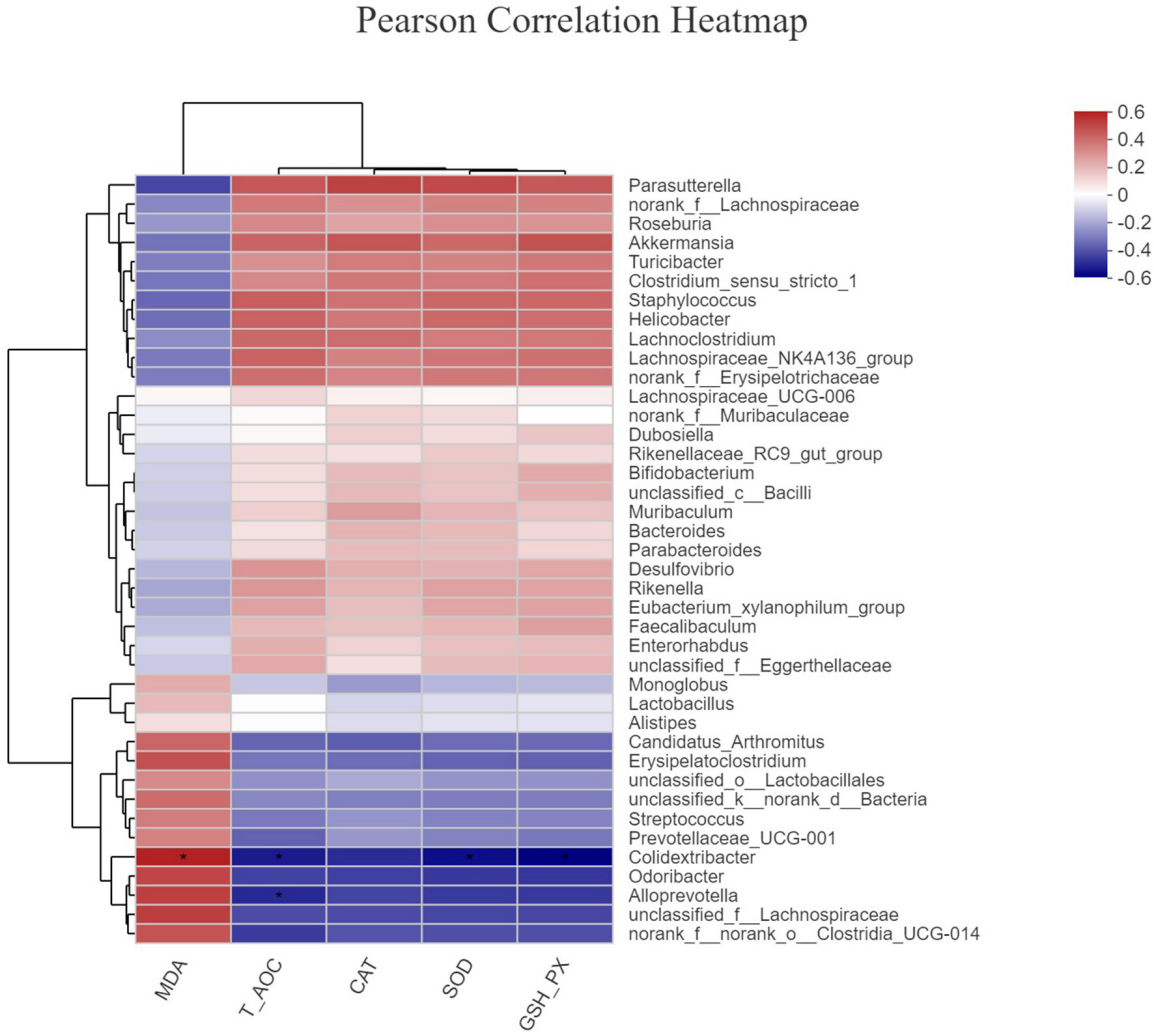
The correlation between the gut microbial population at the genus level and the serum antioxidant indicators. *P < 0.05.

To investigate whether the alteration in gut microbiota is associated with the antioxidant effects of OE, we performed a correlation analysis using the *Firmicutes/Bacteroidetes* ratio and serum antioxidant indicators (T-AOC, SOD, GSH-Px, CAT, and MDA). As shown in Figure 8, there was a negative correlation between the *Firmicutes/Bacteroidetes* ratio and the level of serum MDA (*P* < 0.05; Figure 8E) as well as a positive correlation between the *Firmicutes/Bacteroidetes* ratio and the activity of serum GSH-Px (*P* < 0.05; Figure 8C). Moreover, there was a positive correlation trend between the *Firmicutes/Bacteroidetes* ratio and the activities of serum T-AOC (0.05 < *P* < 0.1), SOD (0.05 < *P* < 0.1), and CAT (0.05 < *P* < 0.1; Figures 8A,B,D). In addition, heat map revealed the correlation between the gut microbial population at the genus level and the serum antioxidant indicators (T-AOC, SOD, GSH-Px, CAT, and MDA). The data showed that the relative abundance of *Colidextribacter* was positively correlated with serum MDA level and negatively correlated with serum T-AOC, SOD, and GSH-Px levels (*P* < 0.05; Figure 9). The relative abundance of *Alloprevotella* was found to be likely markedly negatively correlated with the serum T-AOC (*P* < 0.05; Figure 9).

### OE Supplementation Had No Effects on Colonic SCFAs

**Figure 10:**
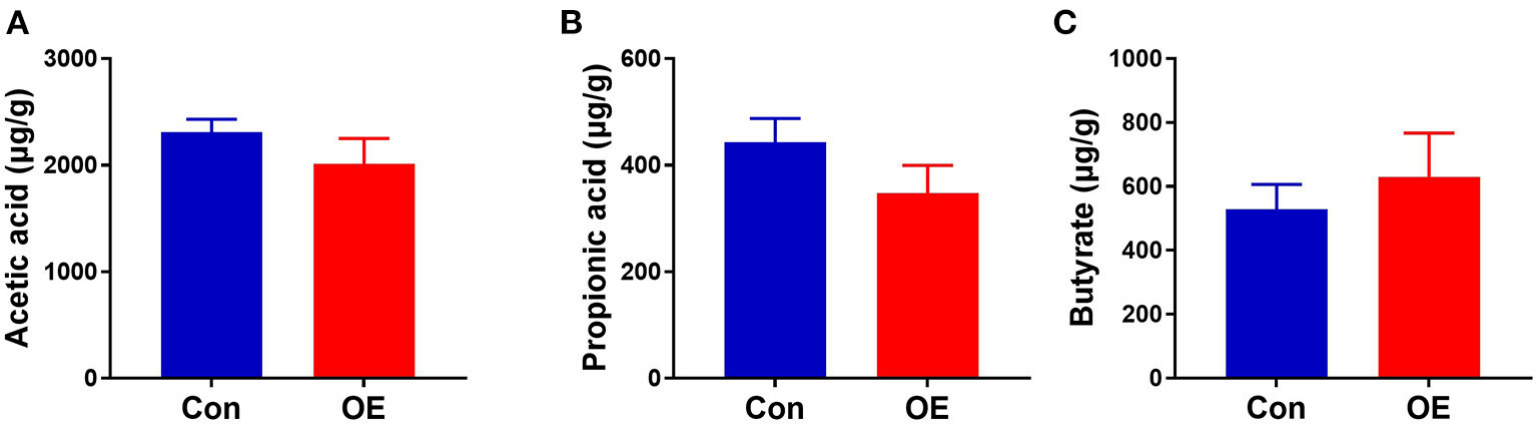
Effects of olive fruit (*Canarium album* L.) extracts on the colonic short-chain fatty acid concentration. **(A)** Acetic acid. **(B)** Propionic acid. **(C)** Butyrate. Data were expressed as mean ± SEM.

Since the supplement of OE altered colonic microbiota composition and structure and SCFAs as the metabolites of microbiota, we investigated the SCFA content in the colon. The results showed that oral administration of OE had no significant effect on the levels of SCFAs, including acetic acid, propionic acid, and butyrate, in the colon (*P* > 0.05; Figure 10).

## Discussion

Studies being conducted both in animal models and humans have revealed the significant role of oxidative stress in the pathogenesis of various diseases, including neurodegenerative and metabolic diseases and cancer (3, 33–35). Emerging evidence has highlighted the beneficial bioactivity of olive fruit, pomace, leaf extracts, and olive oils because of the many bioactive polyphenolic compounds, which have various health-promoting potentials including antioxidant, anti-inflammation, and anti-bacteria (36–38). In this study, we have demonstrated that oral administration of OE was able to enhance the antioxidative capacity as well as the anti-inflammatory activity in mice, which may be associated with the changes of gut microbiota induced by OE treatment.

Investigations have shown that olive extracts contain polyphenols which exhibit powerful antioxidant and anti-inflammatory effects on humans and animals (39, 40). For instance, numerous evidence has demonstrated that olive extracts can ameliorate oxidative stress and inflammation in different cells, such as colon cancer cells (41), kidney cells (42, 43), and renal cells (44). In addition, oral administration of olive extracts could alleviate the lipopolysaccharide (LPS)-induced oxidative stress and inflammatory responses as shown by attenuating the decreased levels of brain GSH and increased levels of brain MDA and serum TNF-α in mice (12). Similarly, in this study, we found that oral administration of OE increased the serum T-AOC and the activities of antioxidant enzymes, including SOD, GSH-Px, and CAT, and decreased the MDA levels in mice. SOD, GSH-Px, and CAT are generally regarded as the primary antioxidant enzyme defense system in animals and humans (45). SOD can catalyze superoxide into oxygen and hydrogen peroxide (45). CAT has the ability to scavenge hydrogen peroxide into oxygen and water (45). GSH-Px can catalyze hydrogen peroxide into water (46). MDA is one of the products of lipid peroxidation, and it is an important indicator of oxidative stress status in the body (47). These results of serum indicators suggested that OE supplement could enhance the antioxidant ability in mice.

The intestine, a vital organ responsible for nutrient digestion and absorption and a major site of host immunity, is highly susceptible to oxidative stress, which leads to gut dysfunction and body disorders (2, 11, 48, 49). It is well-documented that dietary polyphenols can be absorbed in the small intestine (50) and exhibit antioxidant effects by scavenging oxidant chemical species as well as altering the levels and activities of antioxidant enzymes (11). On the other hand, polyphenolic compounds are commonly recognized as xenobiotics by the enterocytes, which will induce stress (51). Thus, we investigated the effects of OE on oxidative stress in the small intestine of mice. Based on the activities of serum antioxidation enzymes, we then detected the transcript levels of Nrf2-associated antioxidant enzymes in the intestine, including *Sod, Cat*, and *Gpx* (52). In mice with severe oxidative stress status, previous studies have shown that extracts from olive oil and olive leaf ameliorated oxidative stress by up-regulating the Nrf2/ARE antioxidant signaling pathways (53, 54). Nrf2, a transcription factor, is activated and translocated to the nucleus during oxidative stress and enhances the expressions of Nrf2-related antioxidant enzymes (55). Interestingly, in this study, the qPCR results showed that OE treatment decreased jejunal and ileal *Sod, Cat, Gpx1*, and *Gpx2* expressions. Consistently, our study also indicated that OE treatment increased the MDA concentration and decreased T-AOC in the jejunum and ileum. We speculated that this may be because OE acted as a xenobiotic, which can induce mild stress in the small intestine of mice (51). However, this slight oxidative stress had no negative effect on the small intestine, which was confirmed by the intestinal morphology without change after OE treatment.

In the meantime, amounts of inflammation-related transcription factors were activated under oxidative stress state, which will initiate the inflammatory process and lead to the increased production of pro-inflammatory cytokines (11). TNF-α and IL-1β are usually considered to be the two key regulators of pro-inflammatory response, which are involved in promoting inflammation and causing tissue damage (56). A previous study has demonstrated that olive extracts had anti-IL-1β activity in humans (57). An *in vitro* study also found that polyphenol-rich olive extracts decreased the mRNA expression of pro-inflammatory cytokines (58). Consistently, in this study, we also found that OE treatment decreased the mRNA expressions of TNF-α and IL-1β in the jejunum and ileum. However, interestingly, we found that the levels of inflammatory cytokines in the small intestine (jejunum and ileum) were all significantly increased, which may be caused by the OE-induced mild oxidative stress. Furthermore, we suggested that the absorption of OE in the small intestine caused down-regulation in mRNA expression of pro-inflammatory cytokines (TNF-α and IL-1β) to play anti-inflammatory activity. Based on the above-mentioned data, we speculated that OE tends to enhance the anti-inflammatory capacity by down-regulating the expressions of pro-inflammatory cytokines.

According to emerging evidence, the majority of dietary polyphenols (including HT) are metabolized by the colonic microbiota (59–61). Meanwhile, *in vivo* studies showed that both olive extracts with complex composition and individual phenolic compounds purified from olive extracts had the ability to modulate bacterial growth and reproduction in the intestine (21, 22). However, little is known about the effects of OE administration on the gut microbiota and whether the gut microbiota associates with the antioxidant and anti-inflammatory effects of OE in mice. Therefore, we analyzed the diversity of the colonic microbial community in OE-treated mice. The results showed that oral administration of OE supplement decreased the Shannon index, suggesting that OE lowered the α-diversity of colonic microbiota, which might be due to the anti-bacterial effects of OE. Additionally, LEfSe results showed that *Staphylococcales* are enriched in the OE group, suggesting that the increased *Staphylococcales* after OE treatment may be one of the reasons for the enhanced antioxidant capacity. Consistently, another study reported that *Staphylococcales* can reduce endogenous and exogenous oxidative stress (62). Besides that, at the phylum level, OE treatment increased the *Firmicutes*/*Bacteroidetes* ratio. As the main metabolites of colonic microbiota, the increased *Firmicutes/Bacteroidetes* ratio did not change the composition of SCFAs significantly in this study, which is in agreement with the previous study (63). To further examine whether OE-induced microbial alteration associates with its antioxidant effects, we conduct correlation analyses between the *Firmicutes*/*Bacteroidetes* ratio and serum antioxidant enzyme activities and MDA level by Pearson correlation analysis. Surprisingly, the results showed that the *Firmicutes*/*Bacteroidetes* ratio was negatively correlated with the serum MDA content and positively correlated with the serum GSH-Px activity. In piglets and sows, studies have indicated that oxidative stress has a direct correlation with gut microbiota (64–67). This information in our study contributed to the new understanding of the OE-enhanced antioxidant capacity, at least in part, due to alterations in the gut microbiota in mice. In addition, *Firmicutes* are considered to be involved in maintaining intestinal barrier integrity, which plays a key role in modulating host inflammation (68). Meanwhile, bacteria in phylum *Bacteroidetes* have the ability to release LPS, which then leads to higher inflammatory responses (69). So, a decreased proportion of *Bacteroidetes* may be related to lower inflammatory factors, which is consistent with our results. In humans, healthy adults who are more resistant to pathogens have a higher *Firmicutes*/*Bacteroidetes* ratio than infants and the elderly (70). Similarly, in piglets, a higher *Firmicutes*/*Bacteroidetes* ratio is associated with enhanced oxidative response and lower inflammation and infection risk (71–73). Therefore, it appears reasonable to speculate that OE administration exhibits antioxidant and anti-inflammatory effects closely associated with the increased *Firmicutes*/*Bacteroidetes* ratio in the colon of mice. At the genus level, *Alloprevotella* was highly negatively correlated with serum T-AOC. Similarly, Zhang et al. showed that OE can ameliorate oxidative stress and reduce the relative abundance of fecal *Alloprevotella* in LPS-challenged piglets (74), suggesting that *Alloprevotella* may play a role in enhancing antioxidant capacity. In addition, we also found that *Colidextribacter* is highly correlated with the levels of MDA, T-AOC, SOD, and GSH-Px in serum, which however needs further investigation.

**Figure 11:**
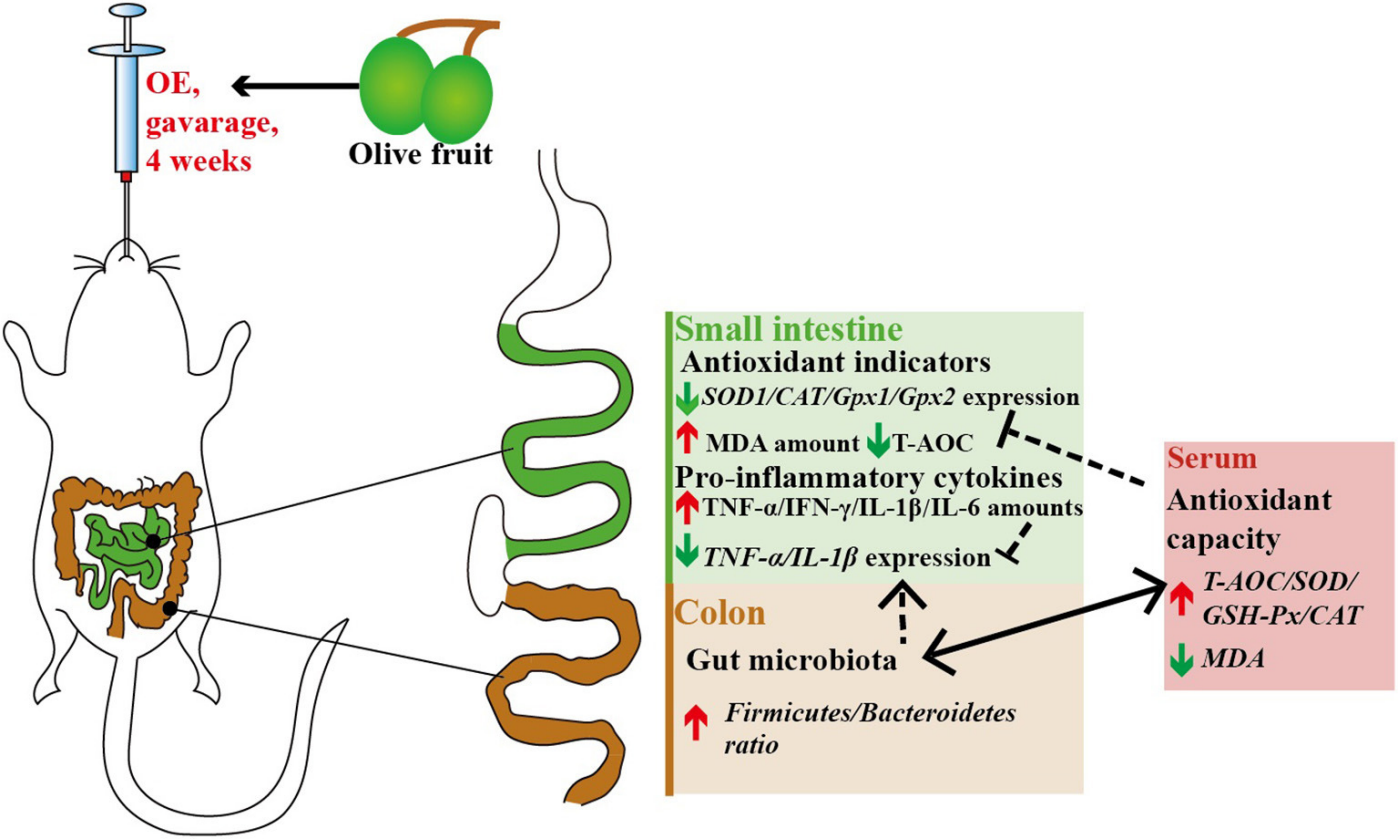
Antioxidant and anti-inflammatory effects of olive fruit (*Canarium album* L.) extracts administration in mice: a possible mechanism.

Although OE contains various polyphenols, current evidence indicates that the beneficial health properties of OE are mainly related to HT, which needs more and further investigation (24–26). As summarized in Figure 11, in conclusion, our data provide a new insight that oral treatment with OE can improve the antioxidant capacity by enhancing the circulating activities of antioxidant enzymes, and the most important is that the improved antioxidant capacity is connected to the increased colonic *Firmicutes*/*Bacteroidetes* ratio as well as the change of *Alloprevotella* and *Colidextribacter* in mice.

## Data Availability Statement

The data used to support the findings of this study are available from the corresponding author upon request. Sequencing raw data on colonic microbiota of mice were deposited into the NCBI Sequence Read Archive (SRA) database (PRJNA681369).

## Ethics Statement

The studies involving human participants were reviewed and approved by Experimental Animal Welfare and Ethical Committee of the Institute of Animal Science, Chinese Academy of Agricultural Sciences. The patients/participants provided their written informed consent to participate in this study. The animal study was reviewed and approved by Experimental Animal Welfare and Ethical Committee of the Institute of Animal Science, Chinese Academy of Agricultural Sciences. Written informed consent was obtained from the owners for the participation of their animals in this study.

## Author Contributions

MW and SZ conducted the study. MW, RZ, SZ, and FW helped to perform the experiment. MW, LC, and RZ helped to write the paper. BY and HZ designed the experiment and revised the manuscript. All authors contributed to the article and approved the submitted version.

## Conflict of Interest

The authors declare that the research was conducted in the absence of any commercial or financial relationships that could be construed as a potential conflict of interest.

## References

[1] Fuccelli R Fabiani R Rosignoli P. Hydroxytyrosol exerts anti-inflammatory and anti-oxidant activities in a mouse model of systemic inflammation. Molecules. (2018) 23:3212. 10.3390/molecules23123212 30563131 PMC6321432

[2] Yin J Liu M Ren W Duan J Yang G Zhao Y. Effects of dietary supplementation with glutamate and aspartate on diquat-induced oxidative stress in piglets. PLoS ONE. (2015) 10:e0122893. 10.1371/journal.pone.0122893 25875335 PMC4398417

[3] Matyas C Haskó G Liaudet L Trojnar E Pacher P. Interplay of cardiovascular mediators, oxidative stress and inflammation in liver disease and its complications. Nat Rev Cardiol. (2021) 18:117–35. 10.1038/s41569-020-0433-5 32999450

[4] He Y Sang Z Zhuo Y Wang X Guo Z He L. Transport stress induces pig jejunum tissue oxidative damage and results in autophagy/mitophagy activation. J Anim Physiol Anim Nutr (Berl). (2019) 103:1521–9. 10.1111/jpn.13161 31328334

[5] Lykkesfeldt J Svendsen O. Oxidants and antioxidants in disease: oxidative stress in farm animals. Vet J. (2007) 173:502–11. 10.1016/j.tvjl.2006.06.005 16914330

[6] Forrester SJ Kikuchi DS Hernandes MS Xu Q Griendling KK. Reactive oxygen species in metabolic and inflammatory signaling. Circ Res. (2018) 122:877–902. 10.1161/circresaha.117.311401 29700084 PMC5926825

[7] Rojo de la Vega M Chapman E Zhang DD. NRF2 and the hallmarks of cancer. Cancer Cell. (2018) 34:21–43. 10.1016/j.ccell.2018.03.022 29731393 PMC6039250

[8] Nguyen M Wong YC Ysselstein D Severino A Krainc D. Synaptic, mitochondrial, and lysosomal dysfunction in Parkinson's disease. Trends Neurosci. (2019) 42:140–9. 10.1016/j.tins.2018.11.001 30509690 PMC6452863

[9] Milic M Frustaci A Del Bufalo A Sánchez-Alarcón J Valencia-Quintana R Russo P. DNA damage in non-communicable diseases: a clinical and epidemiological perspective. Mutat Res. (2015) 776:118–27. 10.1016/j.mrfmmm.2014.11.009 26255943

[10] Mishra V Banga J Silveyra P. Oxidative stress and cellular pathways of asthma and inflammation: therapeutic strategies and pharmacological targets. Pharmacol Ther. (2018) 181:169–82. 10.1016/j.pharmthera.2017.08.011 28842273 PMC5743757

[11] Hussain T Tan B Yin Y Blachier F Tossou MC Rahu N. Oxidative stress and inflammation: what polyphenols can do for us? Oxid Med Cell Longevity. (2016) 2016:7432797. 10.1155/2016/7432797 27738491 PMC5055983

[12] Gupta A Vij G Chopra K. Possible role of oxidative stress and immunological activation in mouse model of chronic fatigue syndrome and its attenuation by olive extract. J Neuroimmunol. (2010) 226:3–7. 10.1016/j.jneuroim.2010.05.021 20537729

[13] Yeh YT Cho YY Hsieh SC Chiang AN. Chinese olive extract ameliorates hepatic lipid accumulation *in vitro* and *in vivo* by regulating lipid metabolism. Sci Rep. (2018) 8:1057. 10.1038/s41598-018-19553-1 29348600 PMC5773498

[14] Erol Ö Arda N Erdem G. Phenols of virgin olive oil protects nuclear DNA against oxidative damage in HeLa cells. Food Chem Toxicol. (2012) 50:3475–9. 10.1016/j.fct.2012.07.048 22877972

[15] Maalej A Mahmoudi A Bouallagui Z Fki I Marrekchi R Sayadi S. Olive phenolic compounds attenuate deltamethrin-induced liver and kidney toxicity through regulating oxidative stress, inflammation and apoptosis. Food Chem Toxicol. (2017) 106:455–65. 10.1016/j.fct.2017.06.010 28595958

[16] Hermans N Van der Auwera A Breynaert A Verlaet A De Bruyne T Van Gaal L. A red yeast rice-olive extract supplement reduces biomarkers of oxidative stress, OxLDL and Lp-PLA(2), in subjects with metabolic syndrome: a randomised, double-blind, placebo-controlled trial. Trials. (2017) 18:302. 10.1186/s13063-017-2058-5 28673363 PMC5496259

[17] Flamminii F Di Mattia CD Difonzo G Neri L Faieta M Caponio F. From by-product to food ingredient: evaluation of compositional and technological properties of olive-leaf phenolic extracts. J Sci Food Agric. (2019) 99:6620–7. 10.1002/jsfa.9949 31350764

[18] Weinbrenner T Fitó M de la Torre R Saez GT Rijken P Tormos C. Olive oils high in phenolic compounds modulate oxidative/antioxidative status in men. J Nutr. (2004) 134:2314–21. 10.1093/jn/134.9.2314 15333722

[19] Çoban J Öztezcan S Dogru-Abbasoglu S Bingül I Yeşil-Mizrak K Uysal M. Olive leaf extract decreases age-induced oxidative stress in major organs of aged rats. Geriatr Gerontol Int. (2014) 14:996–1002. 10.1111/ggi.12192 24854676

[20] De Bruno A Romeo R Fedele FL Sicari A Piscopo A Poiana M. Antioxidant activity shown by olive pomace extracts. J Environ Sci Health B. (2018) 53:526–33. 10.1080/03601234.2018.1462928 29708834

[21] Kishikawa A Ashour A Zhu Q Yasuda M Ishikawa H Shimizu K. Multiple biological effects of olive oil by-products such as leaves, stems, flowers, olive milled waste, fruit pulp, and seeds of the olive plant on skin. Phytother Res. (2015) 29:877–86. 10.1002/ptr.5326 25779104

[22] Gilling DH Ravishankar S Bright KR. Antimicrobial efficacy of plant essential oils and extracts against *Escherichia coli*. J Environ Sci Health A Tox Hazard Subst Environ Eng. (2019) 54:608–16. 10.1080/10934529.2019.1574153 30821189

[23] Almayouf MA El-Khadragy MF Awad MA Al-Olayan EM. The effects of silver nanoparticles biosynthesized using fig and olive extracts on cutaneous leishmaniasis induced inflammation in female Balb/c Mice. Biosci Rep. (2020) 40:BSR20202672. 10.1042/BSR20202672 33252120 PMC7745064

[24] Basiricò L Morera P Dipasquale D Bernini R Santi L Romani A. (-)-Epigallocatechin-3-gallate and hydroxytyrosol improved antioxidative and anti-inflammatory responses in bovine mammary epithelial cells. Animal. (2019) 13:2847–56. 10.1017/s1751731119001356 31182175

[25] Yonezawa Y Miyashita T Nejishima H Takeda Y Imai K Ogawa H. Anti-inflammatory effects of olive-derived hydroxytyrosol on lipopolysaccharide-induced inflammation in RAW264.7 cells. J Vet Med Sci. (2018) 80:1801–7. 10.1292/jvms.18-0250 30298817 PMC6305503

[26] Fonollá J Maldonado-Lobón JA Luque R Rodríguez C Bañuelos Ó López-Larramendi JL. Effects of a combination of extracts from olive fruit and almonds skin on oxidative and inflammation markers in hypercholesterolemic subjects: a randomized controlled trial. J Med Food. (2020). 10.1089/jmf.2020.0088. [Epub ahead of print]. 32816626 PMC8140357

[27] Scoditti E Carpi S Massaro M Pellegrino M Polini B Carluccio MA. Hydroxytyrosol modulates adipocyte gene and miRNA expression under inflammatory condition. Nutrients. (2019) 11:2493. 10.3390/nu11102493 31627295 PMC6836288

[28] Cardona F Andrés-Lacueva C Tulipani S Tinahones FJ Queipo-Ortuño MI. Benefits of polyphenols on gut microbiota and implications in human health. J Nutr Biochem. (2013) 24:1415–22. 10.1016/j.jnutbio.2013.05.001 23849454

[29] Santhakumar AB Battino M Alvarez-Suarez JM. Dietary polyphenols: structures, bioavailability and protective effects against atherosclerosis. Food Chem Toxicol. (2018) 113:49–65. 10.1016/j.fct.2018.01.022 29360556

[30] Conterno L Martinelli F Tamburini M Fava F Mancini A Sordo M. Measuring the impact of olive pomace enriched biscuits on the gut microbiota and its metabolic activity in mildly hypercholesterolaemic subjects. Eur J Nutr. (2019) 58:63–81. 10.1007/s00394-017-1572-2 29124388 PMC6424929

[31] Millman J Okamoto S Kimura A Uema T Higa M Yonamine M. Metabolically and immunologically beneficial impact of extra virgin olive and flaxseed oils on composition of gut microbiota in mice. Eur J Nutr. (2020) 59:2411–25. 10.1007/s00394-019-02088-0 31506767 PMC7413911

[32] Vezza T Rodríguez-Nogales A Algieri F Garrido-Mesa J Romero M Sánchez M. The metabolic and vascular protective effects of olive (*Olea europaea* L.) leaf extract in diet-induced obesity in mice are related to the amelioration of gut microbiota dysbiosis and to its immunomodulatory properties. Pharmacol Res. (2019) 150:104487. 10.1016/j.phrs.2019.104487 31610229

[33] Kahroba H Ramezani B Maadi H Sadeghi MR Jaberie H Ramezani F. The role of Nrf2 in neural stem/progenitors cells: from maintaining stemness and self-renewal to promoting differentiation capability and facilitating therapeutic application in neurodegenerative disease. Ageing Res Rev. (2020) 65:101211. 10.1016/j.arr.2020.101211 33186670

[34] Stern M McNew JA. A transition to degeneration triggered by oxidative stress in degenerative disorders. Mol Psychiatry. (2021) 26:736–46. 10.1038/s41380-020-00943-9 33159186 PMC7914161

[35] Vatner SF Zhang J Oydanich M Berkman T Naftalovich R Vatner DE. Healthful aging mediated by inhibition of oxidative stress. Ageing Res Rev. (2020) 64:101194. 10.1016/j.arr.2020.101194 33091597 PMC7710569

[36] Martín-Vertedor D Garrido M Pariente JA Espino J Delgado-Adámez J. Bioavailability of bioactive molecules from olive leaf extracts and its functional value. Phytother Res. (2016) 30:1172–9. 10.1002/ptr.5625 27137173

[37] Medina E Romero C García P Brenes M. Characterization of bioactive compounds in commercial olive leaf extracts, and olive leaves and their infusions. Food Funct. (2019) 10:4716–24. 10.1039/c9fo00698b 31304950

[38] Gorzynik-Debicka M Przychodzen P Cappello F Kuban-Jankowska A Marino Gammazza A Knap N. Potential health benefits of olive oil and plant polyphenols. Int J Mol Sci. (2018) 19:686. 10.3390/ijms19030686 29495598 PMC5877547

[39] Martínez L Castillo J Ros G Nieto G. Antioxidant and antimicrobial activity of rosemary, pomegranate and olive extracts in fish patties. Antioxidants (Basel). (2019) 8:86. 10.3390/antiox8040086 30987153 PMC6523725

[40] Schaffer S Podstawa M Visioli F Bogani P Müller WE Eckert GP. Hydroxytyrosol-rich olive mill wastewater extract protects brain cells *in vitro* and *ex vivo*. J Agric Food Chem. (2007) 55:5043–9. 10.1021/jf0703710 17530860

[41] Sun L Luo C Liu J. Hydroxytyrosol induces apoptosis in human colon cancer cells through ROS generation. Food Funct. (2014) 5:1909–14. 10.1039/c4fo00187g 24953710

[42] Crupi R Palma E Siracusa R Fusco R Gugliandolo E Cordaro M. Protective effect of hydroxytyrosol against oxidative stress induced by the ochratoxin in kidney cells: *in vitro* and *in vivo* Study. Front Vet Sci. (2020) 7:136. 10.3389/fvets.2020.00136 32296717 PMC7136456

[43] Loru D Incani A Deiana M Corona G Atzeri A Melis MP. Protective effect of hydroxytyrosol and tyrosol against oxidative stress in kidney cells. Toxicol Ind Health. (2009) 25:301–10. 10.1177/0748233709103028 19651801

[44] Martínez-Lara E Peña A Calahorra J Cañuelo A Siles E. Hydroxytyrosol decreases the oxidative and nitrosative stress levels and promotes angiogenesis through HIF-1 independent mechanisms in renal hypoxic cells. Food Funct. (2016) 7:540–8. 10.1039/c5fo00928f 26608793

[45] Lei XG Zhu JH Cheng WH Bao Y Ho YS Redd2i AR. Paradoxical roles of antioxidant enzymes: basic mechanisms and health implications. Physiol Rev. (2016) 96:307–64. 10.1152/physrev.00010.2014 26681794 PMC4839492

[46] He L He T Farrar S Ji L Liu T Ma X. Antioxidants maintain cellular redox homeostasis by elimination of reactive oxygen species. Cell Physiol Biochem. (2017) 44:532–53. 10.1159/000485089 29145191

[47] Chen M Cai W Zhao S Shi L Chen Y Li X. Oxidative stress-related biomarkers in saliva and gingival crevicular fluid associated with chronic periodontitis: a systematic review and meta-analysis. J Clin Periodontol. (2019) 46:608–22. 10.1111/jcpe.13112 30989678

[48] Ahlawat S Asha Sharma KK. Gut-organ axis: a microbial outreach and networking. Lett Appl Microbiol. (2020). 10.1111/lam.13333. [Epub ahead of print]. 32472555

[49] Ringseis R Gessner DK Eder K. The gut-liver axis in the control of energy metabolism and food intake in animals. Annu Rev Anim Biosci. (2020) 8:295–319. 10.1146/annurev-animal-021419-083852 31689373

[50] Fraga CG Croft KD Kennedy DO Tomás-Barberán FA. The effects of polyphenols and other bioactives on human health. Food Funct. (2019) 10:514–28. 10.1039/c8fo01997e 30746536

[51] Gessner DK Ringseis R Eder K. Potential of plant polyphenols to combat oxidative stress and inflammatory processes in farm animals. J Anim Physiol Anim Nutr (Berl). (2017) 101:605–28. 10.1111/jpn.12579 27456323

[52] Zhang H Davies KJA Forman HJ. Oxidative stress response and Nrf2 signaling in aging. Free Radic Biol Med. (2015) 88:314–36. 10.1016/j.freeradbiomed.2015.05.036 26066302 PMC4628850

[53] Marinić J Broznić D Milin C. Preexposure to olive oil polyphenols extract increases oxidative load and improves liver mass restoration after hepatectomy in mice *via* stress-sensitive genes. Oxid Med Cell Longev. (2016) 2016:9191407. 10.1155/2016/9191407 26925195 PMC4746397

[54] Has AL Alotaibi MF Bin-Jumah M Elgebaly H Mahmoud AM. *Olea europaea* leaf extract up-regulates Nrf2/ARE/HO-1 signaling and attenuates cyclophosphamide-induced oxidative stress, inflammation and apoptosis in rat kidney. Biomed Pharmacother. (2019) 111:676–85. 10.1016/j.biopha.2018.12.112 30611992

[55] Omidian K Rafiei H Bandy B. Polyphenol inhibition of benzo[a]pyrene-induced oxidative stress and neoplastic transformation in an *in vitro* model of carcinogenesis. Food Chem Toxicol. (2017) 106:165–74. 10.1016/j.fct.2017.05.037 28533128

[56] Yahfoufi N Alsadi N Jambi M Matar C. The immunomodulatory and anti-inflammatory role of polyphenols. Nutrients. (2018) 10:1618. 10.3390/nu10111618 30400131 PMC6266803

[57] Wauquier F Mevel E Krisa S Richard T Valls J Hornedo-Ortega R. Chondroprotective properties of human-enriched serum following polyphenol extract absorption: results from an exploratory clinical trial. Nutrients. (2019) 11:9071. 10.3390/nu11123071 31888255 PMC6950735

[58] Kountouri AM Kaliora AC Koumbi L Andrikopoulos NK. *In-vitro* gastric cancer prevention by a polyphenol-rich extract from olives through induction of apoptosis. Eur J Cancer Prev. (2009) 18:33–9. 10.1097/CEJ.0b013e3282fb75f7 19077562

[59] Tuck KL Freeman MP Hayball PJ Stretch GL Stupans I. The *in vivo* fate of hydroxytyrosol and tyrosol, antioxidant phenolic constituents of olive oil, after intravenous and oral dosing of labeled compounds to rats. J Nutr. (2001) 131:1993–6. 10.1093/jn/131.7.1993 11435519

[60] Visioli F Galli C Bornet F Mattei A Patelli R Galli G. Olive oil phenolics are dose-dependently absorbed in humans. FEBS Lett. (2000) 468:159–60. 10.1016/s0014-5793(00)01216-3 10692578

[61] D'Angelo S Manna C Migliardi V Mazzoni O Morrica P Capasso G. Pharmacokinetics and metabolism of hydroxytyrosol, a natural antioxidant from olive oil. Drug Metab Dispos. (2001) 29:1492–8. 10.1016/S1359-6446(01)01977-8 11602527

[62] Gaupp R Ledala N Somerville GA. Staphylococcal response to oxidative stress. Front Cell Infect Microbiol. (2012) 2:33. 10.3389/fcimb.2012.00033 22919625 PMC3417528

[63] Giuliani C Marzorati M Daghio M Franzetti A Innocenti M Van de Wiele T. Effects of olive and pomegranate by-products on human microbiota: a study using the SHIME(®) *in vitro* simulator. Molecules. (2019) 24:3791. 10.3390/molecules24203791 31640295 PMC6832639

[64] Wang H Hu C Cheng C Cui J Ji Y Hao X. Unraveling the association of fecal microbiota and oxidative stress with stillbirth rate of sows. Theriogenology. (2019) 136:131–7. 10.1016/j.theriogenology.2019.06.028 31255919

[65] Qiao Y Sun J Ding Y Le G Shi Y. Alterations of the gut microbiota in high-fat diet mice is strongly linked to oxidative stress. Appl Microbiol Biotechnol. (2013) 97:1689–97. 10.1007/s00253-012-4323-6 22948953

[66] Li Y Liu H Zhang L Yang Y Lin Y Zhuo Y. Maternal dietary fiber composition during gestation induces changes in offspring antioxidative capacity, inflammatory response, and gut microbiota in a sow model. Int J Mol Sci. (2019) 21:31. 10.3390/ijms21010031 31861629 PMC6981455

[67] Nie Y Hu J Hou Q Zheng W Zhang X Yang T. Lactobacillus frumenti improves antioxidant capacity *via* nitric oxide synthase 1 in intestinal epithelial cells. Faseb J. (2019) 33:10705–16. 10.1096/fj.201900253RR 31262191

[68] Louis P Flint HJ. Formation of propionate and butyrate by the human colonic microbiota. Environ Microbiol. (2017) 19:29–41. 10.1111/1462-2920.13589 27928878

[69] Ortega-Hernández A Martínez-Martínez E Gómez-Gordo R López-Andrés N Fernández-Celis A Gutiérrrez-Miranda B. The interaction between mitochondrial oxidative stress and gut microbiota in the cardiometabolic consequences in diet-induced obese rats. Antioxidants (Basel). (2020) 9:640. 10.3390/antiox9070640 32708095 PMC7402124

[70] Mariat D Firmesse O Levenez F Guimarăes V Sokol H Doré J. The Firmicutes/Bacteroidetes ratio of the human microbiota changes with age. BMC Microbiol. (2009) 9:123. 10.1186/1471-2180-9-123 19508720 PMC2702274

[71] Chae JP Pajarillo EA Oh JK Kim H Kang DK. Revealing the combined effects of lactulose and probiotic enterococci on the swine faecal microbiota using 454 pyrosequencing. Microb Biotechnol. (2016) 9:486–95. 10.1111/1751-7915.12370 27305897 PMC4919990

[72] Molist F Manzanilla EG Pérez JF Nyachoti CM. Coarse, but not finely ground, dietary fibre increases intestinal Firmicutes:Bacteroidetes ratio and reduces diarrhoea induced by experimental infection in piglets. Br J Nutr. (2012) 108:9–15. 10.1017/s0007114511005216 22018207

[73] Hu R He Z Liu M Tan J Zhang H Hou DX. Dietary protocatechuic acid ameliorates inflammation and up-regulates intestinal tight junction proteins by modulating gut microbiota in LPS-challenged piglets. J Anim Sci Biotechnol. (2020) 11:92. 10.1186/s40104-020-00492-9 32944233 PMC7487840

[74] Yu Z Zhao-Xi D Mao-Long H Xin ML Jian-Xin L Haifeng W. Olive extract ameliorates oxidative stress and inflammation, and protects intestinal villus and microbiota in piglets induced by Lipopolysaccharides. Res Square. (2021). 10.21203/rs.3.rs-101171/v1. [Epub ahead of print].

